# Quantifying the added value of foot-controlled force variables in predicting mild cognitive impairment

**DOI:** 10.1038/s41598-026-62094-1

**Published:** 2026-07-15

**Authors:** Daniel Koska, Andresa Germano, Daniel Schmidt, Ann-Kathrin Harsch, Christian Maiwald

**Affiliations:** Institute of Human Movement Science and Health, Chemnitz University of Technology, 09126 Chemnitz, Germany

**Keywords:** Mild cognitive impairment, Dementia, Prediction, Force control, Complexity, Risk factors, Predictive markers, Dementia, Alzheimer's disease

## Abstract

Predicting early cognitive decline is important for timely interventions. However, existing approaches for early detection remain limited by cost, scalability, or concerns about their validity. This study evaluated the potential of lower-extremity force control capacities, a largely unexplored area, to improve prediction of cognitive status in older adults with and without Mild Cognitive Impairment (MCI). MCI refers to an early stage of cognitive decline that does not meet criteria for dementia. 224 participants aged 80–91 years completed a foot-pedal tracking task in which they continuously adjusted the force applied to a pedal to match a visual target curve presented at two different frequencies. The task was designed to capture ongoing visuomotor monitoring and fine motor control. Task performance was quantified using measures of tracking accuracy (Root Mean Square Error, RMSE) and signal complexity (Sample Entropy, SampEn). These reflect how precisely participants controlled force and how regular their output was over time. Participants with MCI showed lower accuracy and lower complexity than cognitively healthy individuals. Adding RMSE and SampEn to a baseline prediction model improved model performance, with the largest gains observed at the higher curve frequency: RMSE contributed 13% new predictive information, SampEn 17%, and both variables together 21%. These gains indicate that foot-controlled force measures added meaningful information beyond established predictors, although confirmation in independent samples is needed. Foot-controlled force tasks may therefore provide a quick-to-administer, objective complement for early detection of cognitive decline.

## Introduction

Aging is associated with an increased prevalence of cognitive and motor deficits^1^, imposing substantial personal and socioeconomic costs. These challenges are amplified when aging is accompanied by pronounced declines in memory and thinking abilities, as observed in dementia. Dementia remains incurable to date. However, early diagnosis is crucial for initiating interventions aimed at alleviating symptoms, such as developing routines and coping strategies. Early diagnosis also allows for planning future care, understanding preferences, and making necessary legal and financial arrangements.

Early detection of pathological aging processes remains challenging, however, because objective measures such as imaging techniques or blood-based biomarkers are complex, costly, and typically reserved for cases in which symptoms have already manifested. Cognitive tests, such as the Montreal Cognitive Assessment (MoCA)^2^ and the Mini-Mental State Examination^3^, are more practical and cost-effective, making them suitable for regular screening. These tests also underpin the concept of mild cognitive impairment (MCI)^2^. MCI is a stage between normal aging and dementia characterized by measurable cognitive abnormalities with largely preserved daily functioning. Individuals with MCI are at an elevated risk of developing dementia^4^. This makes MCI an early phase in which interventions are more likely to yield positive outcomes than in later stages of dementia.

Despite the well-established value of cognitive tests, questions about their validity remain, driven by issues such as subjectivity, choice of cutoff values, cross-cultural applicability, and reliability^5–8^. In the search for alternative or complementary screening methods for MCI, several studies have demonstrated that motor impairments can precede cognitive decline^9–12^. The association between gross motor skills, such as gait and balance impairments, and MCI is fairly well-documented^13^. For instance, a noted decrease in walking speed during dual cognitive-motor tasks has proven to be a reliable marker of MCI^14^.

While motor impairments can signal cognitive decline, older adults may initially compensate for changes through increased attentional resources and top-down control^15–18^. However, this ability to compensate tends to decline over time. This may be related to a reduced capacity of the brain to engage additional cognitive resources when needed^19,20^, a decreased ability of the brain to adapt and reorganize (reduced neural plasticity)^21,22^, and a reduction in efficient communication between different brain regions (cortical disconnection)^23,24^. These effects may be even more pronounced in individuals with MCI^25^. As compensatory capacity declines, motor tasks requiring precise and fine-tuned control, such as writing or handling small objects, become particularly challenging as they depend on more extensive cortical processing^26,27^. This suggests that fine motor tasks may be more sensitive to early cognitive changes, making them strong candidates as indicators of MCI.

A relationship between upper-extremity fine motor performance and MCI has been reported for tasks such as finger tapping, handwriting, reaching, and the Pegboard test^28,29^, although the association appears weaker than that observed for gait-related measures. Studies on lower-extremity fine motor performance have mainly focused on coordination tasks such as foot tapping. These have yielded inconclusive evidence regarding differences between individuals with MCI and cognitively healthy individuals (CHI)^30–33^.

One comparatively understudied domain of fine motor control is force control. Force control requires precise regulation of muscle force and continuous sensorimotor adjustments. It is therefore expected to place substantial demands on cognitive processes that are vulnerable in individuals with MCI. Compared with coordination tasks, force control may thus represent a more sensitive domain for detecting early cognitive changes. For the upper extremities, studies have shown that measures of force control accuracy and complexity (i.e, signal regularity) can distinguish between older and younger adults^34–39^. These variables reflect different aspects of motor behavior: accuracy primarily captures precision, whereas complexity reflects how the motor control system manages adjustments and responds to changing task demands.

Only few studies have examined force control in relation to MCI. Rudisch et al.^40^ examined a bimanual force control task requiring participants to follow constant or alternating sine-wave target curves but observed only marginal differences in accuracy and complexity. In a similar task involving role-differentiated bimanual movements with the same participants, the authors reported slightly larger differences between MCI and CHI^41^. No comparable lower-extremity studies involving individuals with MCI have been published. This contrasts with the importance of precise foot control for everyday activities involving continuous interaction with the environment, such as driving a car^42^. The closest related evidence comes from Beccherle et al.^43^, who identified foot-eye coordination as a parameter affected by cognitive impairment. Their study, however, focused on moderate Alzheimer’s disease rather than MCI.

To address this gap, we conducted a force control experiment in which participants reproduced sine curves displayed on a screen by operating a rotating foot pedal. We hypothesized that individuals with MCI and CHI differ in both accuracy and complexity of the produced signal, and that these variables can improve the prediction of MCI. We tested this by developing a prediction model incorporating established predictors of MCI and evaluating its performance after adding accuracy and/or complexity variables. We also explored whether task difficulty affects the distinction between MCI and CHI. Specifically, we hypothesized that increasing the sine-curve frequency would further challenge the aforementioned compensatory capacity and thereby improve discrimination between MCI and CHI. These findings may provide new insights into the mechanisms underlying motor control in aging and cognitive impairment, potentially aiding the development of quicker-to-administer, objective screening methods.

## Results

**Fig. 1:**
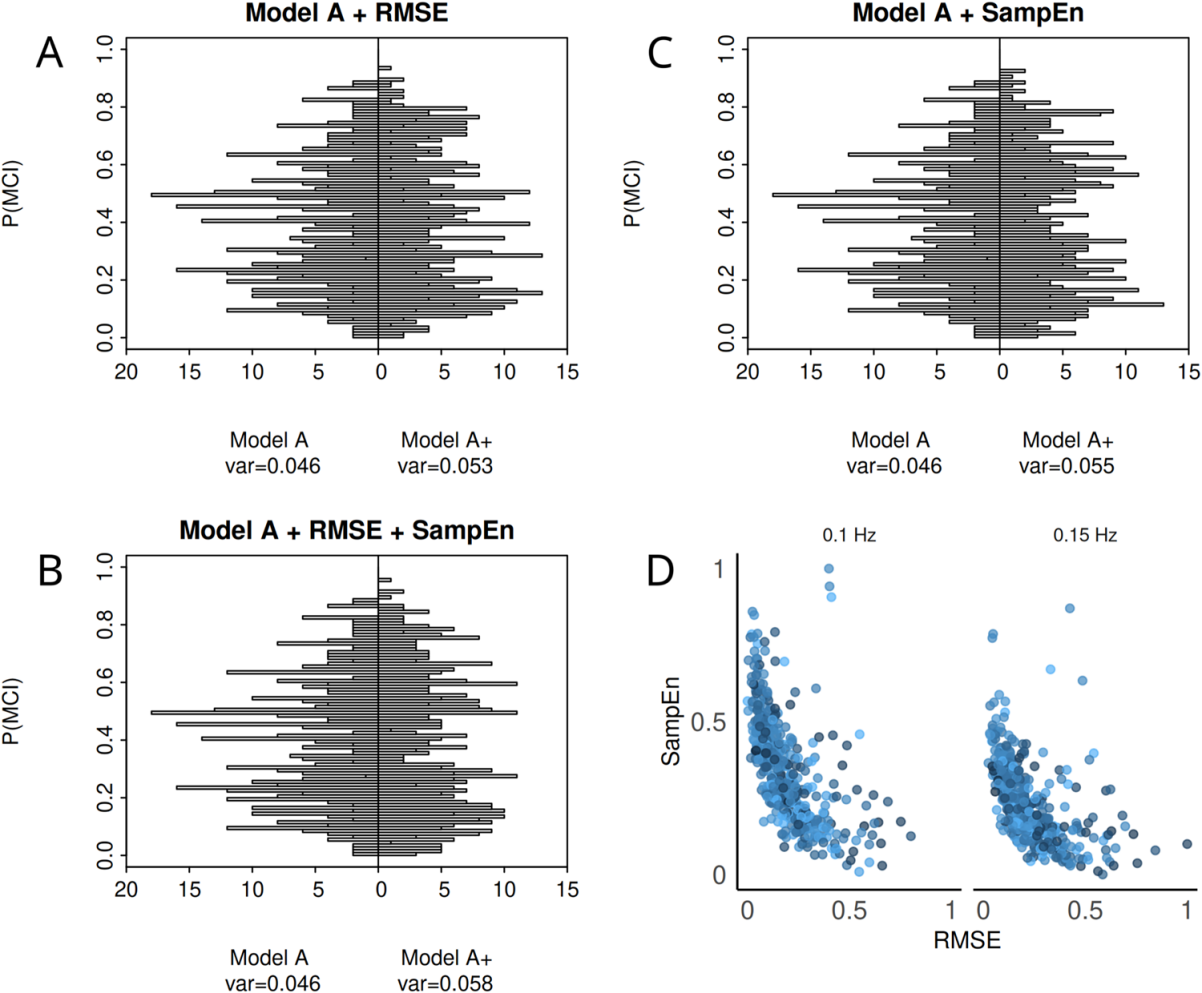
Back-to-back histograms showing predicted probabilities under the baseline model (Model A, left) and the corresponding expanded model (Model A+, right) at a target curve frequency of 0.15 Hz. In panels A–C, Model A+ denotes Model A extended by RMSE, SampEn, or both, respectively. Panel D illustrates the relationship between RMSE and SampEn at both curve frequencies. Darker values indicate lower MoCA scores.

This study evaluated the force control performance of 224 participants aged 80 years and older. Participants applied force to a rotating foot pedal to generate a signal that closely followed a target sine curve at two frequencies (0.1 and 0.15 Hz). Differences between the applied and the target force curve were quantified using measures of accuracy (Root Mean Square Error, RMSE) and complexity (Sample Entropy, SampEn). The added value of these variables in predicting MCI was quantified as the Fraction of New Information (FNI) obtained by incorporating RMSE, SampEn, or both into a baseline logistic regression model (Tab. 2). Predicted probabilities under the baseline model (Model A) and expanded models (Model A extended by RMSE, SampEn, or both; hereafter denoted Model A+) are visualized with back-to-back histograms (Fig. 1). Participant characteristics are presented in Table 1. A graphical distribution of RMSE and SampEn by cognitive group (MCI/CHI) and frequency is presented in Supplementary Fig. S1.

**Table 1:** Participant characteristics and descriptive force-control measures by MoCA group: Cognitively healthy individuals (CHI) and individuals with Mild Cognitive Impairment (MCI; MoCA $\le$ 25).

Variable	CHI	MCI
Participants	135	89
Education (years)	13.8 (3.8)	13.7 (4)
MoCA (points)	27.7 (1.3)	23.3 (1.7)
Age (years)	82.3 (2.4)	82.7 (2.3)
Sex (male/female)	59 / 76	56 / 33
Diabetes (yes/no)	40 / 95	60 / 29
cvd (yes/no)	45 / 90	30 / 59
Activity	0.24 (0.15)	0.26 (0.14)
gds	2.3 (1.0)	2.5 (1.1)
Smoking (yes/no)	10 / 79	20 / 115
Car driving (yes/no)	93 / 42	74 / 15
RMSE	0.19 (0.12)	0.24 (0.16)
SampEn	0.46 (0.16)	0.39 (0.13)

Values are presented as mean (standard deviation) or counts (yes/no). Abbreviations: cvd - cardiovascular disease, gds - geriatric depression score.

Table 2 summarizes the predictive performance of the baseline model and the expanded models at both curve frequencies. Overall, adding force control variables improved model performance, as reflected by higher likelihood ratio chi-square values, higher Nagelkerke’s pseudo R^2^ values, and positive FNI values across all expanded models. The combination of both variables yielded the highest FNI. This improvement was greatest at 0.15 Hz (FNI = 0.21). These findings are consistent with our initial hypothesis that RMSE and SampEn improve discrimination between individuals with MCI and CHI. They further suggest that the added value of these variables increases with task difficulty. This pattern was more pronounced for SampEn, whose FNI more than doubled at the higher frequency.

**Table 2:** Prediction model performance: Likelihood ratio (LR) chi square, Nagelkerkes pseudo R^2^, mean squared error (MSE) between predictions and observations, and Fraction of New Information (FNI) for different expanded models across curve frequencies (0.1 Hz, 0.15 Hz).

Model	LR $\chi ^2$	Nagelkerke R^2^	MSE	FNI
Model A$_{0.1}$	94.6	0.26 (0.18)	0.19 (0.21)	–
Model A$_{0.1}$ + RMSE	104.59	0.28 (0.20)	0.19 (0.21)	0.10
Model A$_{0.1}$ + SampEn	101.71	0.27 (0.20)	0.19 (0.20)	0.07
Model A$_{0.1}$ + RMSE + SampEn	109.55	0.29 (0.20)	0.19 (0.21)	0.14
Model A$_{0.15}$	94.13	0.26 (0.18)	0.19 (0.21)	-
Model A$_{0.15}$ + RMSE	108.04	0.29 (0.20)	0.19 (0.20)	0.13
Model A$_{0.15}$ + SampEn	113.98	0.30 (0.22)	0.18 (0.20)	0.17
Model A$_{0.15}$ + RMSE + SampEn	118.78	0.32 (0.23)	0.18 (0.20)	0.21

Model A represents the baseline model. Values in parentheses are optimism-corrected (via bootstrap).

Figure 1 complements the results in Table 2 by showing how model expansion changes the distribution of predicted MCI probabilities relative to the baseline model at 0.15 Hz. In all three scenarios (adding RMSE, SampEn, or both), the histograms became slightly wider at the upper end of the probability scale. This suggests that expanded models provide greater differentiation among individuals with higher predicted probabilities of MCI. Results for the lower frequency (0.1 Hz) are similar (Supplementary Fig. S2). Panel D shows a pronounced association between RMSE and SampEn, which appears to weaken at higher RMSE values.

Table 3 presents the regression coefficients for the expanded model including both RMSE and SampEn at 0.15 Hz. Among the force-control variables, SampEn showed a positive main coefficient ($\beta$ = 3.75) and a more pronounced negative spline term ($\beta$ = -9.13). In contrast, RMSE coefficients were smaller ($\beta$ = 1.06; $\beta$ = 1.74) and remained positive across both terms. For the lower curve frequency (0.1 Hz, see Supplementary Table S3), most model coefficients were similar, except for RMSE. Here, the main RMSE coefficient was negative (-1.28), in contrast to the positive value at 0.15 Hz. The spline term was also larger at 0.1 Hz than at 0.15 Hz (6.40 vs. 1.74). This suggests that the modeled association of RMSE with MCI differed between frequencies.

**Table 3:** Regression coefficients of the extended logistic regression model at 0.15 Hz.

	Coefficient	Standard Error	Wald Z	Pr(>$\mid$Z$\mid$)
Intercept	−75.78	48.64	−1.56	0.12
Age	0.93	0.61	1.54	0.12
Age′	−31.70	11.24	-2.82	0.01
Age$^{\prime \prime }$	107.15	32.55	3.29	0.00
Age$^{\prime \prime \prime }$	−130.48	34.57	−3.78	0.00
Sex=M	59.54	68.76	0.87	0.39
Diabetes=NO	−0.88	0.35	-2.55	0.01
cvd=NO	−0.07	0.25	-0.30	0.77
Gds	0.20	0.05	4.22	0.00
Activity	0.08	0.78	0.11	0.92
Car driving=NO	−1.05	0.33	-3.17	0.00
Smoking=YES	−1.49	0.37	-4.00	0.00
Side=R	0.09	0.23	0.41	0.68
RMSE	1.06	3.40	0.31	0.76
RMSE$^{\prime }$	1.74	4.26	0.41	0.68
SampEn	3.75	3.06	1.23	0.22
SampEn$^{\prime }$	−9.13	3.85	−2.37	0.02
Age * sex=M	−0.73	0.86	−0.85	0.39
Age′ * sex=M	23.50	15.16	1.55	0.12
Age$^{\prime \prime }$ * sex=M	−81.70	43.22	−1.89	0.06
Age$^{\prime \prime \prime }$ * sex=M	103.21	44.85	2.30	0.02

Coefficients represent estimated changes in the log odds of MCI. For categorical variables, coefficients are interpreted relative to the reference category (e.g., sex = M indicates the effect of being male compared to female; side = R indicates the effect of performing the task with the right foot compared to the left). Wald z values indicate how far the estimated coefficients lie from zero in standard error units. Prime symbols indicate spline terms; asterisks denote interactions. Abbreviations: cvd - cardiovascular disease, gds - geriatric depression score.

**Fig. 2:**
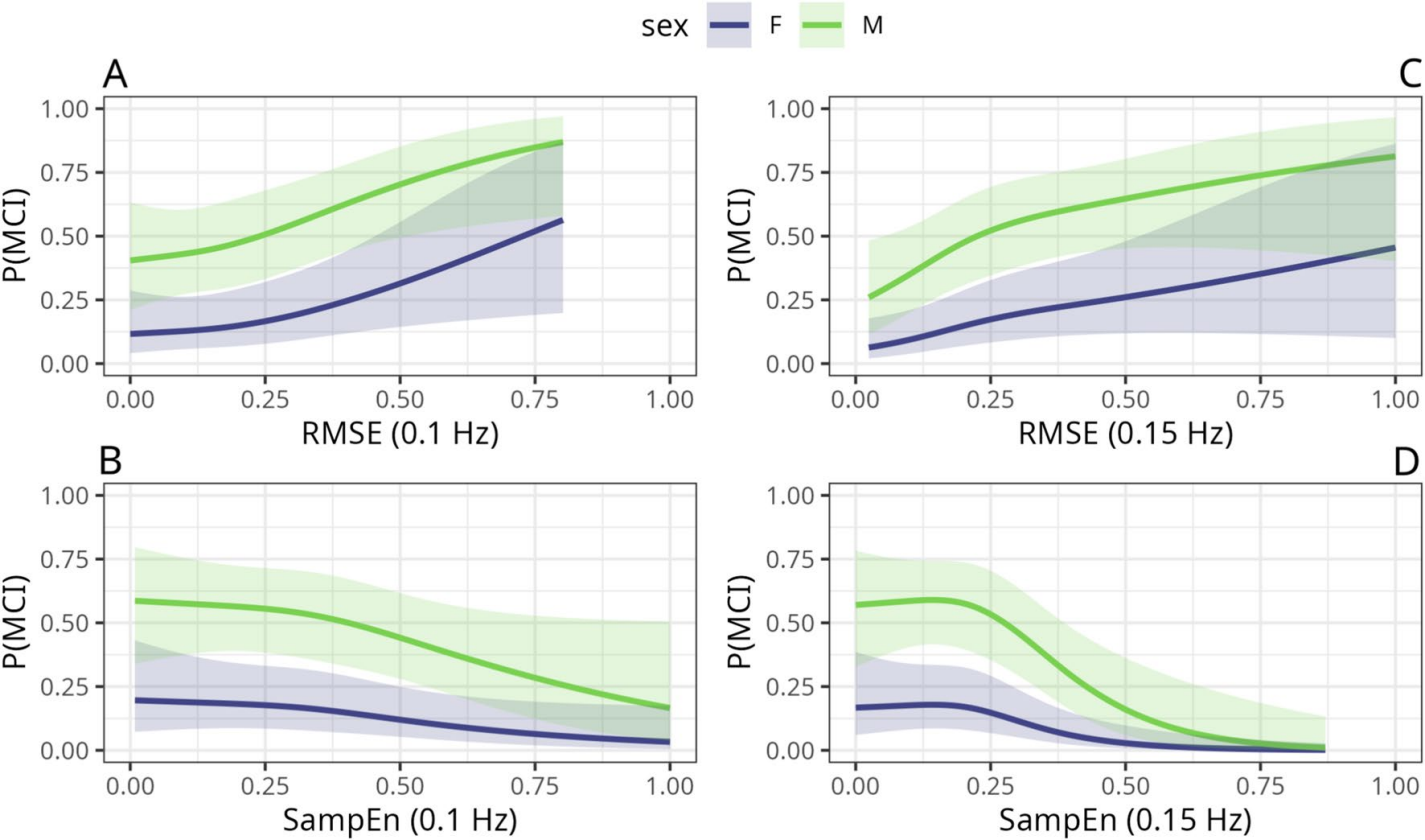
Estimated probability of MCI as a function of RMSE and SampEn for males and females across curve frequencies. Predictions are adjusted for the most frequent category (no diabetes medication, cvd medication, non-smoker, still driving) and medians (e.g., age = 82, depression score = 3, activity score = 0.21). Curves show mean and 95% confidence limits.

Fig. 2 illustrates the predicted probabilities of MCI as functions of RMSE and SampEn at both curve frequencies, stratified by sex. Across all scenarios, males showed higher predicted probabilities of MCI than females. With the exception of SampEn at 0.15 Hz, all curves showed a monotonic relationship with the predicted probability of MCI. Higher RMSE values were consistently associated with higher predicted probabilities of MCI, whereas lower SampEn values were associated with higher predicted probabilities of MCI. Confidence intervals varied across predictors, predictor range, and frequency.

## Discussion

This study investigated whether variables from a lower-extremity fine motor task could improve the prediction of MCI in older adults. Participants used a rotating foot pedal to follow a sine target curve. RMSE and SampEn were derived from the resulting signals and added to a logistic regression baseline model. Both measures improved MCI prediction, as reflected by the Fraction of New Information and high-resolution histograms of predicted probabilities. The improvement was greater at the higher of the two curve frequencies, where RMSE contributed 13% new information, SampEn 17%, and both variables together 21%. These findings suggest that lower-extremity force control variables may provide meaningful added value for predicting MCI.

Descriptively, participants with MCI had higher RMSE values than CHI (Tab. 1). This indicates poorer force control accuracy and is in line with the idea outlined in the introduction that MCI may reduce the capacity to compensate for fine motor deficits. Reductions in force-control accuracy have been observed in various studies comparing older and younger CHI^34,35,37,44^. Studies on force control in MCI, however, remain limited. Two previous investigations based on the same cohort as the present study (SENDA) reported differences in force-control accuracy between CHI and MCI in upper-extremity tasks^40,41^. In both studies, the task type (constant vs. alternating force production) influenced the magnitude of these differences.

Participants with MCI also had lower SampEn values than CHI (Tab. 1). This is consistent with the loss-of-complexity hypothesis, according to which healthy systems show greater complexity and therefore greater adaptability. In motor behavior, this may reflect a broader repertoire of control strategies and a greater capacity to adapt to changing task demands. Similar effects have been described across physiological, cognitive, and motor systems^45–47^. The same two studies based on the SENDA cohort that reported differences in force-control accuracy also examined complexity. In Rudisch et al.^40^, complexity was quantified using the scaling exponent of detrended fluctuation analysis (DFA-$\alpha$). Differences in DFA-$\alpha$ between CHI and MCI were small and statistically non-significant. In Rudisch et al.^41^, older CHI displayed higher complexity (higher SampEn and lower DFA-$\alpha$) than MCI during a bimanual constant task, but lower complexity during a role-differentiated task. These findings suggest that changes in complexity, similar to those in accuracy, depend on task demands. Comparable task-dependent patterns have been reported in studies comparing younger and older adults^35,37^. Comparisons across studies, however, are complicated by the use of different complexity metrics. Compared with metrics such as DFA-$\alpha$, entropy measures more directly quantify signal regularity and predictability, which may be particularly informative in goal-directed motor-control tasks requiring continuous corrective adjustments. Sample entropy in particular is less sensitive to data length than related metrics such as approximate entropy^48,49^. This makes it particularly well suited to the signals analyzed in the present study.

As shown in Fig. 1, RMSE and SampEn are correlated. This indicates that complexity and accuracy are related. However, the association weakens at higher RMSE values, suggesting that the two variables do not reflect the same aspect of motor control^50^. This interpretation is supported by the regression results. SampEn demonstrated a stronger main effect and nonlinear association with MCI, whereas RMSE exhibited smaller coefficients and a sign change in the nonlinear term between frequencies. In addition, including both variables in the same model produced the highest FNI values. Combining measures of force-control accuracy and complexity may therefore better reflect the multifaceted effects of MCI on motor control.

Increasing the curve frequency improved the distinction between MCI and CHI and led to higher FNI values (Tab. 2). This is consistent with the idea that more demanding tasks require greater cortical involvement and may therefore reveal deficits more clearly when compensatory capacity is reduced^17^. Similar frequency-dependent effects have been reported in upper-extremity force-control studies. Here, varying curve frequencies caused different responses across age groups^35,37^. Task difficulty may also be optimized in other ways, for instance by using more complex trajectories, bilateral tasks^41^, or dual-task conditions^51,52^. Such manipulations may help identify task settings that are particularly sensitive to early cognitive changes.

The validity of the estimated FNIs depends substantially on the quality of the baseline model. Our baseline logistic regression model included age and sex, as well as key variables from several relevant domains, such as health conditions and lifestyle factors. Other potentially relevant predictors, such as social interaction, diet, sleep, drug use, or stress levels^53^, were either unavailable or did not sufficiently improve model performance to justify additional model complexity. This is especially relevant because the optimism-corrected performance metrics (Table 2) already indicate some degree of overfitting. In addition, while several regression coefficients were broadly in line with expectations, others were not. For example, the model suggested a lower probability of MCI among smokers. This contradicts both existing literature^54,55^ and intuitive reasoning. Likewise, a lower probability of MCI was predicted for participants who no longer drove a car. Physical activity showed no discernible association with MCI status. These findings may reflect sparse data in certain categories, loss of information due to dichotomization^56–58^, or limited validity of some available covariates. Nevertheless, the baseline model performed reasonably well, with a Nagelkerke R^2^ of 0.26 and an MSE of 0.19. This suggests that it provides a useful basis for evaluating the added value of the force-control variables.

The interpretation of the model performance should also take into account the uncertainty of the outcome itself. MCI status was based on MoCA scores, and MoCA is known to show within-person variability^8^. In our sample, 66 participants completed the MoCA four times over a two-year period, and the mean within-person range was 3.5 points. This degree of variation necessarily introduces uncertainty into the outcome classification and limits the explanatory power of any model fitted to it.

Another source of uncertainty is the cutoff used to distinguish MCI from CHI. Several studies suggest that the originally proposed threshold of MoCA $\le$ 25^2^ may be too high^7,59^. We therefore repeated the analysis using a lower cutoff of MoCA $\le$ 24. This did not affect SampEn, but it substantially increased the FNI for RMSE (see Supplementary Table S4). At the same time, the previously observed frequency-dependent differences in FNI disappeared for the RMSE-only model and for the model combining RMSE and SampEn. This pattern supports our earlier interpretation that accuracy and complexity may partly reflect different aspects of motor control while also highlighting the sensitivity of the results to the chosen classification threshold.

The sensitivity of some findings to the chosen cutoff also highlights a more general issue, namely whether dichotomizing cognitive scores is appropriate. We used a binary MCI outcome because the originally proposed cutoff^2^ is widely used and because our primary aim was to quantify the added value of lower-extremity force-control variables for distinguishing between participants with and without MCI. Dichotomization, however, inevitably entails loss of information and may reduce statistical efficiency^58,60^. For clinical decision making, models that retain more of the original score information, such as ordinal logistic regression, may therefore be preferable.

Lastly, the generalizability of our findings to other effectors, such as the upper extremities, warrants consideration. From a motor-control and neurocognitive perspective, one might expect similar overall patterns in other effectors. Existing literature on upper-limb motor performance, including in tasks involving visuomotor control, is broadly compatible with this view^28,29^. The present lower-extremity task nevertheless yielded a more consistent distinction between CHI and MCI than the upper-extremity studies conducted in the same participants^40,41^. This comparison, however, should be interpreted with caution. The present study used a unilateral visuomotor foot-pedal sine-tracking task, whereas the upper-extremity SENDA studies involved bimanual hand force-control with different coordination demands. It is therefore possible that differences in task design influenced the detectability of group differences. Future studies aiming to clarify potential effector-specific effects would therefore benefit from implementing comparable experimental paradigms for the upper and lower extremities. Given that the present study is, to our knowledge, the first to analyze sine-wave tracking tasks using foot-controlled force signals in participants with MCI, replication of these findings is an important prerequisite for meaningful cross-effector comparisons.

## Methods

This study was conducted as part of the Sensor-based Systems for Early Detection of Dementia (SENDA) project at Chemnitz University of Technology, registered in the German Clinical Trials Register (DRKS00013167). A comprehensive description of the project, including details on the study design and sample size calculation, is given in the SENDA study protocol^61^.

### Participants and cognitive screening

This study was conducted within the SENDA cohort. For the parent SENDA study, an a priori sample size calculation assuming small to moderate effect sizes ($\alpha$ = 0.05, power = 0.80) indicated a required sample of 200 participants, with a planned recruitment target of 240 participants to account for expected dropout^61^. A total of 227 participants completed the force control task. After excluding one participant due to implausible force curves and two due to missing data, the final sample comprised 224 participants.

Participants were eligible if they were 80 years or older, could visit the lab independently or with assistance, were able to walk unaided, had sufficient German language skills, passed hearing and vision screenings, and resided in the Chemnitz area. Exclusion criteria were: (1) medically prescribed ban from physical activity; (2) diagnosis of psychological or neurocognitive disorder (MoCA score < 19); (3) permanent impairment due to a stroke or brain surgery; (4) other neurological diseases, such as epilepsy, Parkinson, or neuropathy; (5) severe diseases of the cardiovascular, respiratory, or musculoskeletal system; (6) diabetes with diagnosed neuropathy; (7) substance abuse; (8) participation in other clinical studies. Informed consent was obtained from all subjects.

Cognitive status was screened using the German version of the MoCA score^2^. MoCA scores were adjusted for education (+1 point for 12 or more years of education) and the cutoff point between CHI and MCI was set to MCI $\le$ 25^2^. Due to indications that lower thresholds may enhance sensitivity in detecting MCI, the analysis was repeated at a lower cutoff threshold (see Supplementary Table S4).

### Foot-controlled force

**Fig. 3:**
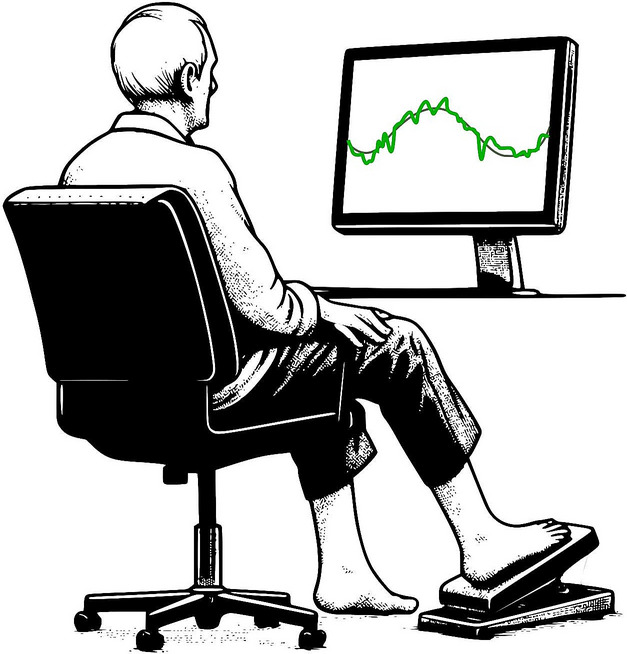
Measurement setup.

Participants performed a seated force-control task in which they rotated a foot pedal to reproduce a sine curve displayed on a screen located approximately 1 m in front of them (Fig. 3). The task was performed separately with the left and right foot. The pedals were equipped with a gas spring (Febrotec, Nitrider 0GS-N06AAA0050, Halver, Germany) and a linear potentiometer (Vishay Electronic GmbH, 249FGJS0XB25, 1 k$\Omega$, Landshut, Germany). The torque required to move the pedal within the operating range was approximately 0.94 Nm.

**Fig. 4:**
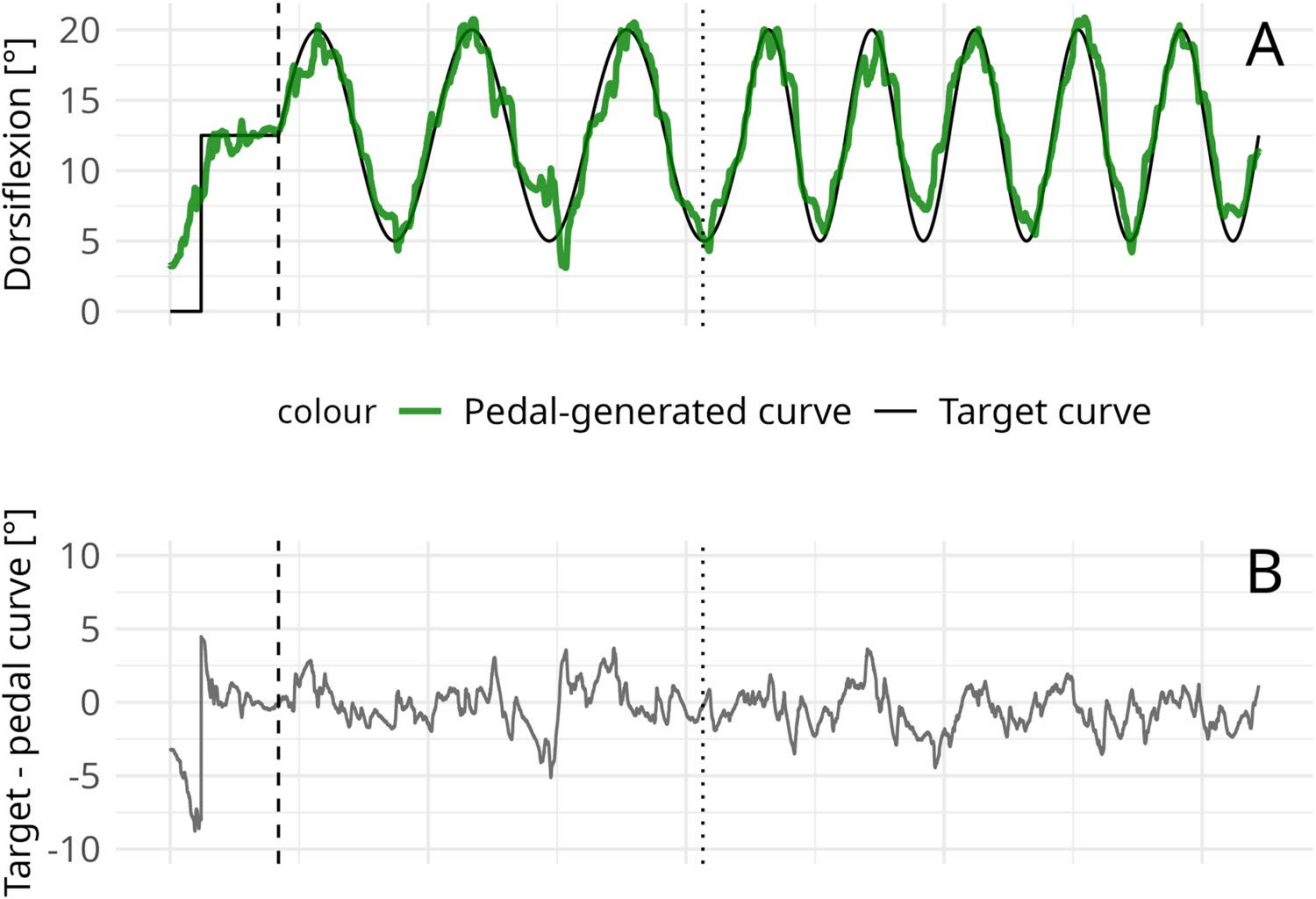
Foot-controlled force curves of an exemplary participant. Upper panel (A): Target curve (black) and pedal-generated force curve (green). The pedal curve was presented as live feedback to the participants. The dashed vertical line indicates the start of the first cycle. The dotted vertical line separates the two curve frequencies (left: 0.1 Hz, right: 0.15 Hz). Lower panel (B): Difference between target and pedal curves.

The goal was to follow the target curve as accurately as possible by rotating the foot pedal appropriately. The target curve consisted of a sine wave with two different frequencies (0.1 Hz and 0.15 Hz) (Fig. 4). Higher modulation frequencies were tested in pilot trials but were excluded as they promoted the emergence of a simple internally generated rhythm, reducing the need for continuous visually guided force adjustments. The 0.1 Hz condition consisted of three cycles, whereas the 0.15 Hz condition consisted of five cycles.

In the starting position, the pedal was tilted 45$^\circ$ with respect to the floor and could be freely rotated in either direction (± 20$^\circ$). The test started when the pedal was pressed. The experiment included three trials per foot in randomized order. Prior to the test, a single practice trial per foot was performed. Data were recorded at a sampling rate of 100 Hz. Each trial lasted approximately 1 min.

### Data processing

Raw force data were not filtered or otherwise processed, but several trials were excluded: The trials of two participants were excluded due to premature measurement starts detected during a visual plausibility check. Only the first of three trials per participant was included to eliminate potential learning effects. The initial part of the signal up until the start of the first cycle was excluded to eliminate artifacts caused by participants missing the start of the measurement.

### Measures of task performance

For the calculation of task performance variables, three cycles of each frequency were included and time-normalized to ensure equal signal lengths. These correspond to the entire signal in the 0.1 Hz condition and to the first three cycles in the 0.15 Hz condition (Fig. 4). Two task performance variables were computed from these signals using the difference between the participant-generated pedal curves and the target curve (Fig. 4, bottom):

1. *Root Mean Square Error (RMSE)* The RMSE calculates the average of the squared differences between the target and participant-generated curves over the task duration, measuring precision and consistency in replicating the force profile. Lower RMSE values indicate greater accuracy and adherence to the target.
2. *Sample Entropy (SampEn)* SampEn was used to quantify the complexity or regularity of the curve differences. SampEn is a modified version of the Approximate Entropy^62^, designed to be more robust to time series length^48,49^. These entropy measures quantify the negative natural logarithm of the conditional probability that a short sequence of length *m* is repeated within the time series^48^. Repeated means that other sequences are available that are arbitrarily similar, i.e., within a specified tolerance *r* and continue to exhibit similarity at the subsequent point. As suggested in^63^, different parameter constellations for *m* and *r* were examined. The input parameters for the function *SampEn*(*m*, *r*, *N*) were then set to *m* = 2 and *r* = 0.3. *N* corresponds to the number of data points. The implementation was done using the R package *pracma*^64^. SampEn is typically a non-negative value, ranging from 0 upwards. Higher values indicate more irregularity or complexity in the data.

### Statistical analysis

From a range of available variables across different domains (e.g., anthropometry, health conditions, lifestyle factors), several regression models were developed and compared using the Akaike Information Criterion. From this evaluation process, the following model was selected as our baseline model:

$$\begin{aligned} \textit{MCI} \sim \textit{rcs}(\textit{age, 5})*\textit{sex} + \textit{diabetes} + \textit{cvd} + \textit{gds} + \textit{activity} + \textit{car} + \textit{smoking} + \textit{side}, \end{aligned}$$

where the dependent binary variable *MCI* indicates MoCA-based cognitive status (MCI or CHI). $rcs(\text {\textit{age, 5}})$ represents a restricted cubic spline transformation of the variable *age* with five knots. The asterisk indicates an interaction effect between the *age* and *sex* (male/female). Variables with categories in brackets are categorical. Further predictor variables are:

- *Diabetes* (yes/no)
- *Cvd* (cardiovascular disease: yes/no)
- *gds* (geriatric depression scale score: 0-15, where 0 indicates no depression^65^)
- *Activity* (Outcome score of the modified German version of the Baecke Physical Activity Questionnaire^66^)
- *Car* (still driving: yes/no)
- *Smoking* (yes/no)
- *Side* (left (L)/right (R))

*diabetes* and *cvd* indicate if medication for treating these conditions was taken within the last six months. *smoking* distinguishes between smokers and non-smokers (never smoked or had not smoked for 40 years or more, based on items from^67^). For *activity*, metabolic equivalent scores were transformed to energy expenditure per week by multiplying with the body weight. *side* indicates which foot was used in a given trial and reflects anatomical side rather than foot dominance.

Continuous variables *RMSE*, *SampEn*, and *activity* were scaled to a range of 0-1 to match the scale of binary variables, thereby enhancing the interpretability of the regression coefficients. This was achieved by transforming the variables to their minimum and maximum range to standardize their values. The final model was examined for collinearity among the predictors, revealing variance inflation factors close to 1, which indicates little to no multicollinearity.

Likelihood ratio chi square (LR) tests were employed to quantify the additional value of adding RMSE and/or SampEn to the baseline prediction model for MCI. The LR tests compared two binary logistic regression models:

1. Model A (baseline model)
2. Model A+_0.1, 0.15_ (expanded): Model A + *rcs*(*RMSE, 3*) and/or *rcs*(*SampEn, 3*),

where *rcs* restricted cubic splines with default knot locations to accommodate nonlinear relationships^68^. The expanded models were fit separately for each curve frequency using the *rms* package in R^69^. Statistical significance for the two-sided LR tests was evaluated using an alpha level of 0.05.

From the LR test, the Fraction of New information (FNI)^68^ was calculated to quantify the proportion of variation explained by the additional force control variables:

$$\begin{aligned} FNI = 1 - (LR_{Model A} / LR_{Model A+}) \end{aligned}$$

A value of FNI=0 indicates no gain in information. The closer FNI is to 1, the more new information is provided by the expanded model. The FNI statistic is complemented by high-resolution histograms of pre- and post-test probabilities. Assuming well calibrated models, more discriminative models result in wider histograms^68^. Model performance was assessed using the Mean Squared Error (MSE) to quantify the prediction error and Nagelkerke’s pseudo R^2^ to measure the goodness of fit. Both measures were optimism corrected via bootstrap (B=500 iterations) to estimate the model’s generalizability. To illustrate the behavior of the force control variables at different frequencies, separate LR tests were conducted for RMSE and SampEn at each frequency. All statistical analysis was performed in R, version 4.3.3^70^.

## Supplementary Information

Supplementary Information.

## Data Availability

The datasets analyzed in the present study are not publicly available but are available from the corresponding author on reasonable request.
