# Supplementary material for: Quantifying the added value of foot-controlled force variables in predicting mild cognitive impairment
Source: Sci Rep. 2026 Jul 15;16:22261. doi: 10.1038/s41598-026-62094-1 (PMC13372807; doi:10.1038/s41598-026-62094-1)
Supplement: Supplementary file 1 — Supplementary Information. [file 41598_2026_62094_MOESM1_ESM.pdf]

Supplementary Information

Quantifying the added value of foot-controlled force variables in predicting mild cognitive impairment

Daniel Koska, Andresa Germano, Daniel Schmidt, Ann-Kathrin Harsch, Christian Maiwald

Supplementary Figure S1: Distribution of RMSE and SampEn by cognitive group and frequency.

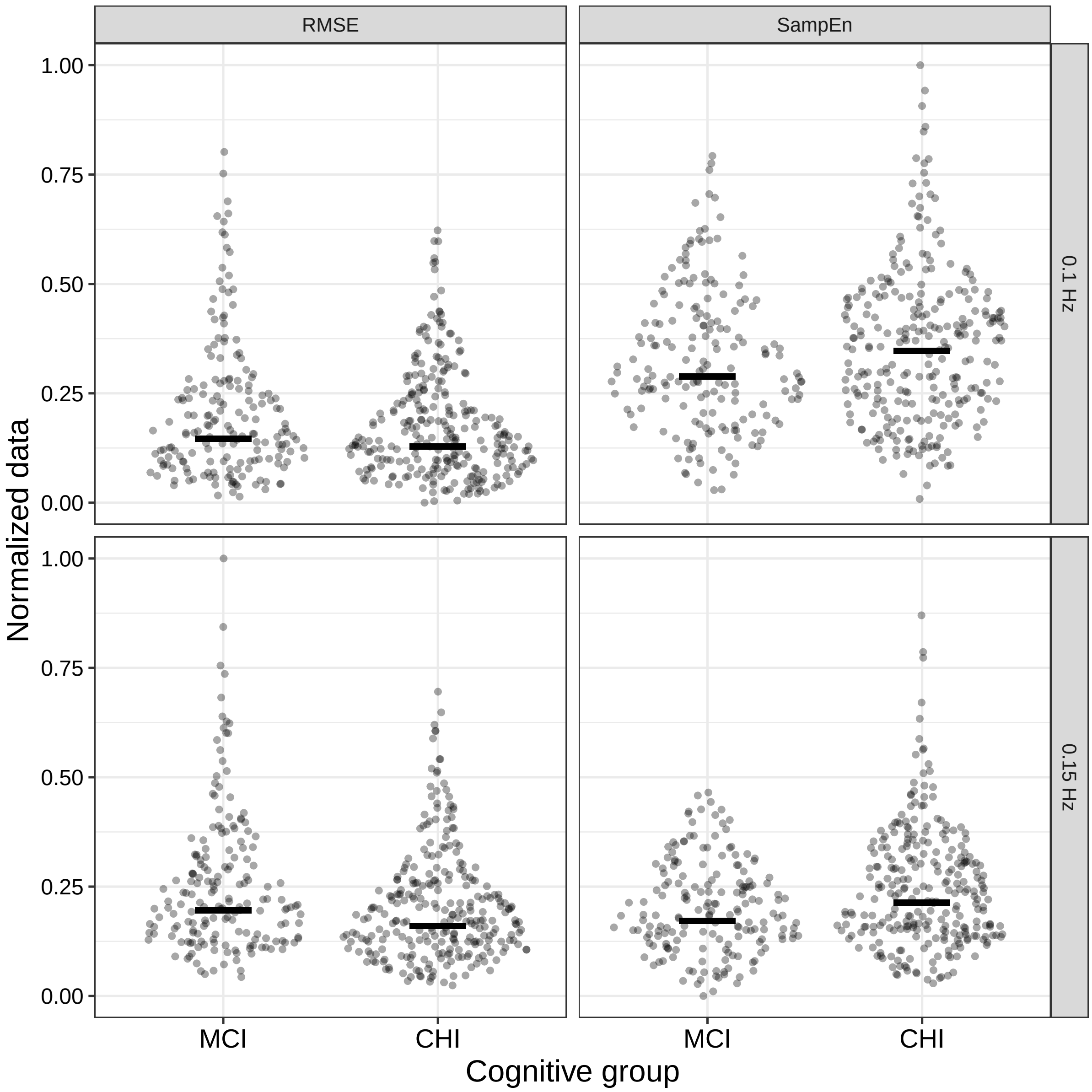

Supplementary Figure S2: Back-to-back histograms of predicted probabilities under the baseline model (Model A, left side) and the expanded models (Model A+, right side) for the lower target curve frequency (0.1 Hz).

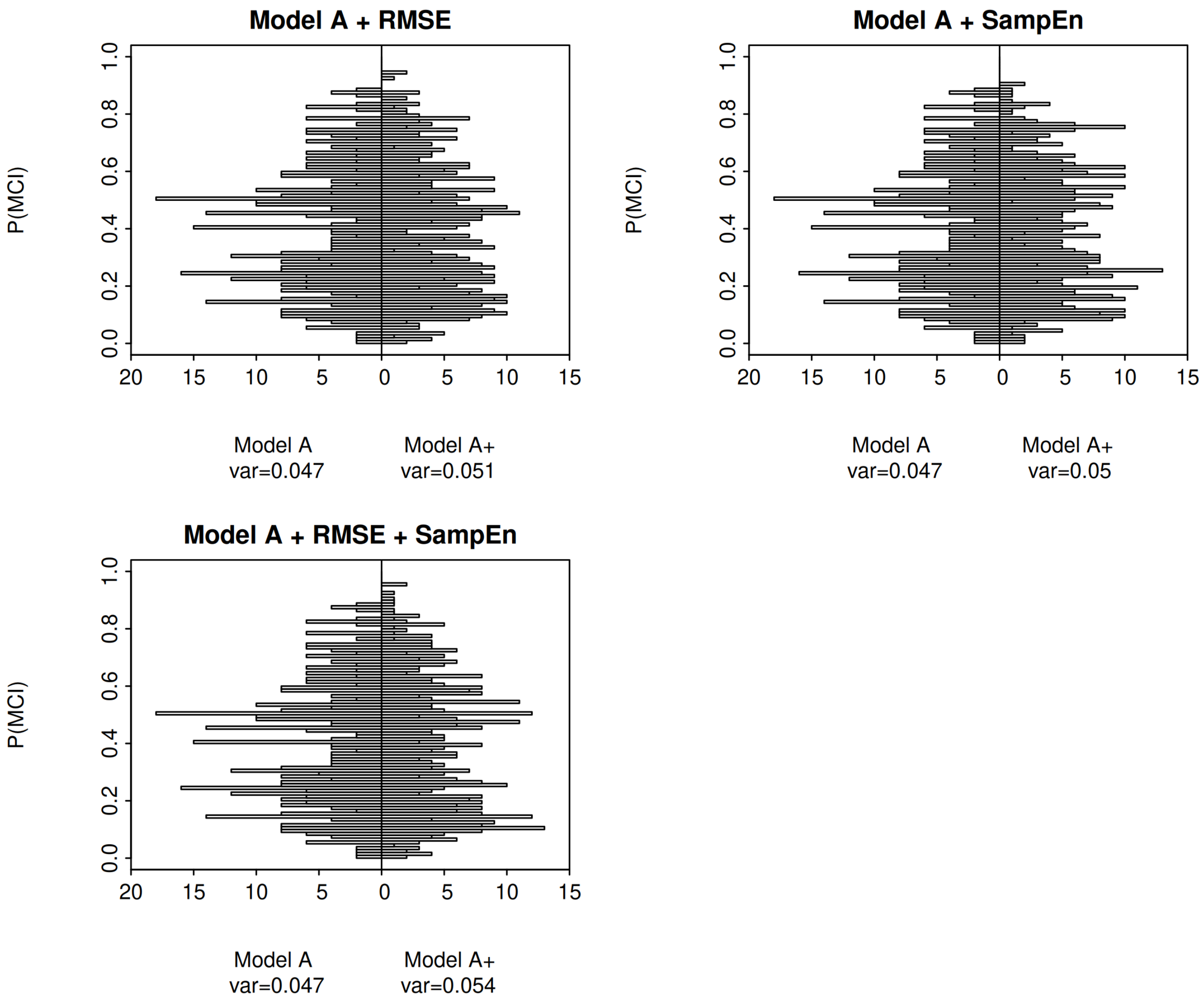

Supplementary Table S3: Summary of the extended logistic regression model including both RMSE and SampEn at 0.1 Hz.

Coefficients represent the estimated change in the log odds of the outcome (MCI). For categorical variables, coefficients indicate the change in log odds of MCI for the specified category relative to the reference level: e.g., sex=M indicates the effect of being male compared to the reference category female. The Wald Z statistic tests the significance of each coefficient by assessing how many standard errors the coefficient is from zero. Acronyms are detailed in the methods section. Prime symbols indicate the order of the spline term, and asterisks indicate interactions.

	**Coefficient**	**Standard Error**	**Wald Z**	**Pr(>|Z|)**
Intercept	-78.85	49.01	-1.61	0.11
age	0.98	0.61	1.60	0.11
age′	-32.20	11.29	-2.85	0.00
age″	109.35	32.72	3.34	0.00
age‴	-133.95	34.75	-3.85	0.00
sex=M	51.41	68.14	0.76	0.45
diabetes=NO	-0.98	0.35	-2.84	0.00
cvd=NO	-0.20	0.24	-0.84	0.40
gds	0.18	0.05	3.95	0.00
activity	0.13	0.77	0.17	0.86
car driving=NO	-1.18	0.33	-3.54	0.00
smoking=YES	-1.37	0.37	-3.74	0.00
side=R	0.10	0.23	0.44	0.66
RMSE	-1.28	3.43	-0.37	0.71
RMSE′	6.40	4.82	1.33	0.18
SampEn	2.45	2.16	1.13	0.26
SampEn′	-4.16	2.25	-1.85	0.07
age * sex=M	-0.63	0.85	-0.74	0.46
age′ * sex=M	22.97	15.08	1.52	0.13
age″ * sex=M	-82.32	43.02	-1.91	0.06
age‴ * sex=M	106.92	44.69	2.39	0.02

Supplementary Table(s) S4: Sensitivity to MoCA cutoff thresholds

The following supplementary tables present model performance metrics using a lower cutoff threshold (MCI $\leq$ 24) than in the main article (MCI $\leq$ 25) for the three investigated scenarios of adding variables to a baseline prediction model (RMSE, SampEn, both). As in the main article, all values are provided for two curve frequencies: 0.1 Hz and 0.15 Hz.

Baseline model (Model A) + RMSE

**Cutoff**	**Frequency**	**Model**	**LR χ^2**	**Nagelkerke R2**	**MSE**	**FNI**
24	0.1	Model A	77.90	0.23 (0.16)	0.16 (0.18)	-
24	0.1	Model A+	97.93	0.29 (0.20)	0.15 (0.17)	0.20
24	0.15	Model A	77.88	0.23 (0.16)	0.16 (0.18)	-
24	0.15	Model A+	98.59	0.29 (0.20)	0.15 (0.17)	0.21

Baseline model (Model A) + SampEn

**Cutoff**	**Frequency**	**Model**	**LR χ^2**	**Nagelkerke R2**	**MSE**	**FNI**
24	0.1	Model A	77.90	0.23 (0.16)	0.16 (0.18)	-
24	0.1	Model A+	82.74	0.25 (0.16)	0.16 (0.18)	0.06
24	0.15	Model A	77.88	0.23 (0.16)	0.16 (0.18)	-
24	0.15	Model A+	92.29	0.27 (0.18)	0.16 (0.17)	0.16

Baseline model (Model A) + RMSE + SampEn

**Cutoff**	**Frequency**	**Model**	**LR χ^2**	**Nagelkerke R2**	**MSE**	**FNI**
24	0.1	Model A	77.90	0.23 (0.16)	0.16 (0.18)	-
24	0.1	Model A+	104.52	0.30 (0.21)	0.15 (0.17)	0.25
24	0.15	Model A	77.88	0.23 (0.16)	0.16 (0.18)	-
24	0.15	Model A+	103.85	0.30 (0.21)	0.15 (0.17)	0.25
